# Supplementary material for: Pyruvate Dehydrogenase Kinase 1 inhibition mediated oxidative phosphorylation enhancement in cartilage promotes osteoarthritis progression
Source: BMC Musculoskelet Disord. 2023 Jul 20;24:597. doi: 10.1186/s12891-023-06585-6 (PMC10357736; doi:10.1186/s12891-023-06585-6)
Supplement: Supplementary file 1 — Additional file 1. [file 12891_2023_6585_MOESM1_ESM.zip › KEGG.Gsea.1653622432464/KEGG_SPLICEOSOME.html]

Details for gene set KEGG\_SPLICEOSOME[GSEA]

|  || Dataset | 55457\_collapsed\_to\_symbols.cls.cls #OA\_versus\_normal.cls.cls #OA\_versus\_normal\_repos |
| Phenotype | cls.cls#OA\_versus\_normal\_repos |
| Upregulated in class | normal |
| GeneSet | KEGG\_SPLICEOSOME |
| Enrichment Score (ES) | -0.2968018 |
| Normalized Enrichment Score (NES) | -1.3245994 |
| Nominal p-value | 0.03894081 |
| FDR q-value | 0.27063376 |
| FWER p-Value | 0.999 |
Table: GSEA Results Summary

  

Fig 1: Enrichment plot: KEGG\_SPLICEOSOME      
 Profile of the Running ES Score & Positions of GeneSet Members on the Rank Ordered List

  

| SYMBOL | TITLE | RANK IN GENE LIST | RANK METRIC SCORE | RUNNING ES | CORE ENRICHMENT || 1 | LSM4 | "LSM4 homolog, U6 small nuclear RNA and mRNA degradation associated [Source:HGNC Symbol;Acc:HGNC:17259]" | 174 | 0.837 | 0.0077 | No |
| 2 | PCBP1 | poly(rC) binding protein 1 [Source:HGNC Symbol;Acc:HGNC:8647] | 197 | 0.823 | 0.0270 | No |
| 3 | TXNL4A | thioredoxin like 4A [Source:HGNC Symbol;Acc:HGNC:30551] | 321 | 0.736 | 0.0362 | No |
| 4 | SF3B5 | splicing factor 3b subunit 5 [Source:HGNC Symbol;Acc:HGNC:21083] | 328 | 0.734 | 0.0544 | No |
| 5 | PPIE | peptidylprolyl isomerase E [Source:HGNC Symbol;Acc:HGNC:9258] | 334 | 0.733 | 0.0727 | No |
| 6 | LSM3 | "LSM3 homolog, U6 small nuclear RNA and mRNA degradation associated [Source:HGNC Symbol;Acc:HGNC:17874]" | 377 | 0.716 | 0.0877 | No |
| 7 | HSPA2 | heat shock protein family A (Hsp70) member 2 [Source:HGNC Symbol;Acc:HGNC:5235] | 662 | 0.603 | 0.0808 | No |
| 8 | CRNKL1 | crooked neck pre-mRNA splicing factor 1 [Source:HGNC Symbol;Acc:HGNC:15762] | 803 | 0.568 | 0.0844 | No |
| 9 | PRPF18 | pre-mRNA processing factor 18 [Source:HGNC Symbol;Acc:HGNC:17351] | 836 | 0.559 | 0.0961 | No |
| 10 | SRSF9 | serine and arginine rich splicing factor 9 [Source:HGNC Symbol;Acc:HGNC:10791] | 882 | 0.548 | 0.1066 | No |
| 11 | DDX23 | DEAD-box helicase 23 [Source:HGNC Symbol;Acc:HGNC:17347] | 913 | 0.538 | 0.1180 | No |
| 12 | USP39 | ubiquitin specific peptidase 39 [Source:HGNC Symbol;Acc:HGNC:20071] | 991 | 0.519 | 0.1252 | No |
| 13 | SNRPD3 | small nuclear ribonucleoprotein D3 polypeptide [Source:HGNC Symbol;Acc:HGNC:11160] | 1046 | 0.508 | 0.1339 | No |
| 14 | HSPA1L | heat shock protein family A (Hsp70) member 1 like [Source:HGNC Symbol;Acc:HGNC:5234] | 1139 | 0.488 | 0.1392 | No |
| 15 | SNRPD2 | small nuclear ribonucleoprotein D2 polypeptide [Source:HGNC Symbol;Acc:HGNC:11159] | 1290 | 0.460 | 0.1392 | No |
| 16 | BCAS2 | BCAS2 pre-mRNA processing factor [Source:HGNC Symbol;Acc:HGNC:975] | 1356 | 0.449 | 0.1455 | No |
| 17 | BUD31 | BUD31 homolog [Source:HGNC Symbol;Acc:HGNC:29629] | 1380 | 0.446 | 0.1551 | No |
| 18 | PPIH | peptidylprolyl isomerase H [Source:HGNC Symbol;Acc:HGNC:14651] | 1555 | 0.419 | 0.1522 | No |
| 19 | SNRPA | small nuclear ribonucleoprotein polypeptide A [Source:HGNC Symbol;Acc:HGNC:11151] | 1670 | 0.403 | 0.1536 | No |
| 20 | SYF2 | SYF2 pre-mRNA splicing factor [Source:HGNC Symbol;Acc:HGNC:19824] | 1881 | 0.375 | 0.1467 | No |
| 21 | SRSF8 | serine and arginine rich splicing factor 8 [Source:HGNC Symbol;Acc:HGNC:16988] | 2053 | 0.356 | 0.1424 | No |
| 22 | LSM5 | "LSM5 homolog, U6 small nuclear RNA and mRNA degradation associated [Source:HGNC Symbol;Acc:HGNC:17162]" | 2127 | 0.347 | 0.1455 | No |
| 23 | SF3A1 | splicing factor 3a subunit 1 [Source:HGNC Symbol;Acc:HGNC:10765] | 3152 | 0.235 | 0.0715 | No |
| 24 | PUF60 | poly(U) binding splicing factor 60 [Source:HGNC Symbol;Acc:HGNC:17042] | 3237 | 0.227 | 0.0707 | No |
| 25 | SNRPF | small nuclear ribonucleoprotein polypeptide F [Source:HGNC Symbol;Acc:HGNC:11162] | 3509 | 0.201 | 0.0546 | No |
| 26 | LSM7 | "LSM7 homolog, U6 small nuclear RNA and mRNA degradation associated [Source:HGNC Symbol;Acc:HGNC:20470]" | 3627 | 0.191 | 0.0504 | No |
| 27 | SNRPC | small nuclear ribonucleoprotein polypeptide C [Source:HGNC Symbol;Acc:HGNC:11157] | 3649 | 0.190 | 0.0536 | No |
| 28 | SNU13 | small nuclear ribonucleoprotein 13 [Source:HGNC Symbol;Acc:HGNC:7819] | 3795 | 0.178 | 0.0468 | No |
| 29 | LSM6 | "LSM6 homolog, U6 small nuclear RNA and mRNA degradation associated [Source:HGNC Symbol;Acc:HGNC:17017]" | 4019 | 0.154 | 0.0333 | No |
| 30 | SNRNP27 | small nuclear ribonucleoprotein U4/U6.U5 subunit 27 [Source:HGNC Symbol;Acc:HGNC:30240] | 4037 | 0.152 | 0.0358 | No |
| 31 | MAGOH | "mago homolog, exon junction complex subunit [Source:HGNC Symbol;Acc:HGNC:6815]" | 4266 | 0.131 | 0.0213 | No |
| 32 | PRPF6 | pre-mRNA processing factor 6 [Source:HGNC Symbol;Acc:HGNC:15860] | 4567 | 0.108 | 0.0006 | No |
| 33 | LSM2 | "LSM2 homolog, U6 small nuclear RNA and mRNA degradation associated [Source:HGNC Symbol;Acc:HGNC:13940]" | 4641 | 0.101 | -0.0025 | No |
| 34 | PRPF4 | pre-mRNA processing factor 4 [Source:HGNC Symbol;Acc:HGNC:17349] | 5076 | 0.067 | -0.0347 | No |
| 35 | CDC40 | cell division cycle 40 [Source:HGNC Symbol;Acc:HGNC:17350] | 5228 | 0.054 | -0.0452 | No |
| 36 | SNRPB2 | small nuclear ribonucleoprotein polypeptide B2 [Source:HGNC Symbol;Acc:HGNC:11155] | 5266 | 0.050 | -0.0468 | No |
| 37 | PRPF8 | pre-mRNA processing factor 8 [Source:HGNC Symbol;Acc:HGNC:17340] | 5287 | 0.049 | -0.0471 | No |
| 38 | CDC5L | cell division cycle 5 like [Source:HGNC Symbol;Acc:HGNC:1743] | 5460 | 0.036 | -0.0596 | No |
| 39 | SNW1 | SNW domain containing 1 [Source:HGNC Symbol;Acc:HGNC:16696] | 5472 | 0.035 | -0.0596 | No |
| 40 | DHX8 | DEAH-box helicase 8 [Source:HGNC Symbol;Acc:HGNC:2749] | 5531 | 0.030 | -0.0634 | No |
| 41 | SNRPE | small nuclear ribonucleoprotein polypeptide E [Source:HGNC Symbol;Acc:HGNC:11161] | 5551 | 0.029 | -0.0641 | No |
| 42 | SART1 | "spliceosome associated factor 1, recruiter of U4/U6.U5 tri-snRNP [Source:HGNC Symbol;Acc:HGNC:10538]" | 5690 | 0.017 | -0.0745 | No |
| 43 | DDX46 | DEAD-box helicase 46 [Source:HGNC Symbol;Acc:HGNC:18681] | 5744 | 0.012 | -0.0783 | No |
| 44 | PQBP1 | polyglutamine binding protein 1 [Source:HGNC Symbol;Acc:HGNC:9330] | 5827 | 0.007 | -0.0845 | No |
| 45 | SF3B2 | splicing factor 3b subunit 2 [Source:HGNC Symbol;Acc:HGNC:10769] | 5895 | 0.002 | -0.0897 | No |
| 46 | SF3A2 | splicing factor 3a subunit 2 [Source:HGNC Symbol;Acc:HGNC:10766] | 5999 | -0.004 | -0.0977 | No |
| 47 | CHERP | calcium homeostasis endoplasmic reticulum protein [Source:HGNC Symbol;Acc:HGNC:16930] | 6081 | -0.011 | -0.1037 | No |
| 48 | SF3B3 | splicing factor 3b subunit 3 [Source:HGNC Symbol;Acc:HGNC:10770] | 6372 | -0.033 | -0.1256 | No |
| 49 | CTNNBL1 | catenin beta like 1 [Source:HGNC Symbol;Acc:HGNC:15879] | 6493 | -0.042 | -0.1339 | No |
| 50 | SF3B4 | splicing factor 3b subunit 4 [Source:HGNC Symbol;Acc:HGNC:10771] | 6777 | -0.061 | -0.1544 | No |
| 51 | LSM8 | "LSM8 homolog, U6 small nuclear RNA associated [Source:HGNC Symbol;Acc:HGNC:20471]" | 6828 | -0.065 | -0.1567 | No |
| 52 | SNRNP200 | small nuclear ribonucleoprotein U5 subunit 200 [Source:HGNC Symbol;Acc:HGNC:30859] | 7249 | -0.099 | -0.1870 | No |
| 53 | WBP11 | WW domain binding protein 11 [Source:HGNC Symbol;Acc:HGNC:16461] | 7312 | -0.103 | -0.1892 | No |
| 54 | HNRNPC | heterogeneous nuclear ribonucleoprotein C [Source:HGNC Symbol;Acc:HGNC:5035] | 7437 | -0.114 | -0.1960 | No |
| 55 | DDX42 | DEAD-box helicase 42 [Source:HGNC Symbol;Acc:HGNC:18676] | 7447 | -0.114 | -0.1938 | No |
| 56 | MAGOHB | "mago homolog B, exon junction complex subunit [Source:HGNC Symbol;Acc:HGNC:25504]" | 7506 | -0.119 | -0.1953 | No |
| 57 | EIF4A3 | eukaryotic translation initiation factor 4A3 [Source:HGNC Symbol;Acc:HGNC:18683] | 7646 | -0.130 | -0.2029 | No |
| 58 | AQR | aquarius intron-binding spliceosomal factor [Source:HGNC Symbol;Acc:HGNC:29513] | 7654 | -0.131 | -0.2001 | No |
| 59 | HSPA1A | heat shock protein family A (Hsp70) member 1A [Source:HGNC Symbol;Acc:HGNC:5232] | 7876 | -0.146 | -0.2136 | No |
| 60 | PRPF31 | pre-mRNA processing factor 31 [Source:HGNC Symbol;Acc:HGNC:15446] | 7898 | -0.147 | -0.2115 | No |
| 61 | HNRNPM | heterogeneous nuclear ribonucleoprotein M [Source:HGNC Symbol;Acc:HGNC:5046] | 8127 | -0.165 | -0.2251 | No |
| 62 | NCBP2 | nuclear cap binding protein subunit 2 [Source:HGNC Symbol;Acc:HGNC:7659] | 8478 | -0.192 | -0.2476 | No |
| 63 | HNRNPA3 | heterogeneous nuclear ribonucleoprotein A3 [Source:HGNC Symbol;Acc:HGNC:24941] | 8603 | -0.201 | -0.2521 | No |
| 64 | RBM8A | RNA binding motif protein 8A [Source:HGNC Symbol;Acc:HGNC:9905] | 8662 | -0.205 | -0.2514 | No |
| 65 | XAB2 | XPA binding protein 2 [Source:HGNC Symbol;Acc:HGNC:14089] | 8778 | -0.215 | -0.2550 | No |
| 66 | RBMX | RNA binding motif protein X-linked [Source:HGNC Symbol;Acc:HGNC:9910] | 8938 | -0.227 | -0.2616 | No |
| 67 | SNRPG | small nuclear ribonucleoprotein polypeptide G [Source:HGNC Symbol;Acc:HGNC:11163] | 9067 | -0.237 | -0.2656 | No |
| 68 | SNRPB | small nuclear ribonucleoprotein polypeptides B and B1 [Source:HGNC Symbol;Acc:HGNC:11153] | 9085 | -0.239 | -0.2608 | No |
| 69 | HSPA8 | heat shock protein family A (Hsp70) member 8 [Source:HGNC Symbol;Acc:HGNC:5241] | 9140 | -0.243 | -0.2588 | No |
| 70 | NCBP1 | nuclear cap binding protein subunit 1 [Source:HGNC Symbol;Acc:HGNC:7658] | 9149 | -0.244 | -0.2533 | No |
| 71 | TRA2B | transformer 2 beta homolog [Source:HGNC Symbol;Acc:HGNC:10781] | 9243 | -0.251 | -0.2541 | No |
| 72 | SNRNP40 | small nuclear ribonucleoprotein U5 subunit 40 [Source:HGNC Symbol;Acc:HGNC:30857] | 9295 | -0.256 | -0.2516 | No |
| 73 | SNRPD1 | small nuclear ribonucleoprotein D1 polypeptide [Source:HGNC Symbol;Acc:HGNC:11158] | 9579 | -0.281 | -0.2666 | No |
| 74 | SMNDC1 | survival motor neuron domain containing 1 [Source:HGNC Symbol;Acc:HGNC:16900] | 9638 | -0.287 | -0.2638 | No |
| 75 | HNRNPU | heterogeneous nuclear ribonucleoprotein U [Source:HGNC Symbol;Acc:HGNC:5048] | 9847 | -0.306 | -0.2722 | No |
| 76 | U2SURP | U2 snRNP associated SURP domain containing [Source:HGNC Symbol;Acc:HGNC:30855] | 10130 | -0.334 | -0.2858 | No |
| 77 | SRSF1 | serine and arginine rich splicing factor 1 [Source:HGNC Symbol;Acc:HGNC:10780] | 10272 | -0.348 | -0.2879 | Yes |
| 78 | SRSF3 | serine and arginine rich splicing factor 3 [Source:HGNC Symbol;Acc:HGNC:10785] | 10280 | -0.349 | -0.2796 | Yes |
| 79 | SRSF5 | serine and arginine rich splicing factor 5 [Source:HGNC Symbol;Acc:HGNC:10787] | 10282 | -0.349 | -0.2708 | Yes |
| 80 | SF3A3 | splicing factor 3a subunit 3 [Source:HGNC Symbol;Acc:HGNC:10767] | 10311 | -0.352 | -0.2640 | Yes |
| 81 | RBM22 | RNA binding motif protein 22 [Source:HGNC Symbol;Acc:HGNC:25503] | 10345 | -0.355 | -0.2575 | Yes |
| 82 | DHX15 | DEAH-box helicase 15 [Source:HGNC Symbol;Acc:HGNC:2738] | 10498 | -0.372 | -0.2599 | Yes |
| 83 | SRSF2 | serine and arginine rich splicing factor 2 [Source:HGNC Symbol;Acc:HGNC:10783] | 10507 | -0.374 | -0.2510 | Yes |
| 84 | HSPA6 | heat shock protein family A (Hsp70) member 6 [Source:HGNC Symbol;Acc:HGNC:5239] | 10561 | -0.379 | -0.2455 | Yes |
| 85 | SRSF6 | serine and arginine rich splicing factor 6 [Source:HGNC Symbol;Acc:HGNC:10788] | 10659 | -0.389 | -0.2431 | Yes |
| 86 | U2AF1 | U2 small nuclear RNA auxiliary factor 1 [Source:HGNC Symbol;Acc:HGNC:12453] | 10762 | -0.400 | -0.2409 | Yes |
| 87 | RBM25 | RNA binding motif protein 25 [Source:HGNC Symbol;Acc:HGNC:23244] | 10800 | -0.405 | -0.2335 | Yes |
| 88 | SRSF10 | serine and arginine rich splicing factor 10 [Source:HGNC Symbol;Acc:HGNC:16713] | 10926 | -0.422 | -0.2325 | Yes |
| 89 | SRSF7 | serine and arginine rich splicing factor 7 [Source:HGNC Symbol;Acc:HGNC:10789] | 10930 | -0.422 | -0.2220 | Yes |
| 90 | SF3B1 | splicing factor 3b subunit 1 [Source:HGNC Symbol;Acc:HGNC:10768] | 10948 | -0.424 | -0.2125 | Yes |
| 91 | PRPF38B | pre-mRNA processing factor 38B [Source:HGNC Symbol;Acc:HGNC:25512] | 11147 | -0.450 | -0.2165 | Yes |
| 92 | SNRPA1 | small nuclear ribonucleoprotein polypeptide A' [Source:HGNC Symbol;Acc:HGNC:11152] | 11218 | -0.459 | -0.2102 | Yes |
| 93 | PRPF19 | pre-mRNA processing factor 19 [Source:HGNC Symbol;Acc:HGNC:17896] | 11269 | -0.467 | -0.2023 | Yes |
| 94 | DHX38 | DEAH-box helicase 38 [Source:HGNC Symbol;Acc:HGNC:17211] | 11860 | -0.562 | -0.2341 | Yes |
| 95 | TCERG1 | transcription elongation regulator 1 [Source:HGNC Symbol;Acc:HGNC:15630] | 11950 | -0.583 | -0.2262 | Yes |
| 96 | ACIN1 | apoptotic chromatin condensation inducer 1 [Source:HGNC Symbol;Acc:HGNC:17066] | 12059 | -0.609 | -0.2191 | Yes |
| 97 | SNRNP70 | small nuclear ribonucleoprotein U1 subunit 70 [Source:HGNC Symbol;Acc:HGNC:11150] | 12073 | -0.613 | -0.2044 | Yes |
| 98 | U2AF2 | U2 small nuclear RNA auxiliary factor 2 [Source:HGNC Symbol;Acc:HGNC:23156] | 12226 | -0.652 | -0.1997 | Yes |
| 99 | HSPA1B | heat shock protein family A (Hsp70) member 1B [Source:HGNC Symbol;Acc:HGNC:5233] | 12278 | -0.670 | -0.1866 | Yes |
| 100 | HNRNPK | heterogeneous nuclear ribonucleoprotein K [Source:HGNC Symbol;Acc:HGNC:5044] | 12341 | -0.688 | -0.1739 | Yes |
| 101 | HNRNPA1 | heterogeneous nuclear ribonucleoprotein A1 [Source:HGNC Symbol;Acc:HGNC:5031] | 12356 | -0.693 | -0.1573 | Yes |
| 102 | DHX16 | DEAH-box helicase 16 [Source:HGNC Symbol;Acc:HGNC:2739] | 12414 | -0.712 | -0.1436 | Yes |
| 103 | TRA2A | transformer 2 alpha homolog [Source:HGNC Symbol;Acc:HGNC:16645] | 12479 | -0.740 | -0.1298 | Yes |
| 104 | THOC1 | THO complex 1 [Source:HGNC Symbol;Acc:HGNC:19070] | 12503 | -0.749 | -0.1125 | Yes |
| 105 | PRPF40A | pre-mRNA processing factor 40 homolog A [Source:HGNC Symbol;Acc:HGNC:16463] | 12651 | -0.826 | -0.1029 | Yes |
| 106 | THOC2 | THO complex 2 [Source:HGNC Symbol;Acc:HGNC:19073] | 12691 | -0.855 | -0.0842 | Yes |
| 107 | SRSF4 | serine and arginine rich splicing factor 4 [Source:HGNC Symbol;Acc:HGNC:10786] | 12749 | -0.911 | -0.0654 | Yes |
| 108 | DDX39B | DExD-box helicase 39B [Source:HGNC Symbol;Acc:HGNC:13917] | 12783 | -0.941 | -0.0440 | Yes |
| 109 | DDX5 | DEAD-box helicase 5 [Source:HGNC Symbol;Acc:HGNC:2746] | 12834 | -1.038 | -0.0214 | Yes |
| 110 | PRPF3 | pre-mRNA processing factor 3 [Source:HGNC Symbol;Acc:HGNC:17348] | 12837 | -1.040 | 0.0049 | Yes |
Table: GSEA details [plain text format]

  

Fig 2: KEGG\_SPLICEOSOME      
 Blue-Pink O' Gram in the Space of the Analyzed GeneSet

  

Fig 3: KEGG\_SPLICEOSOME: Random ES distribution      
 Gene set null distribution of ES for **KEGG\_SPLICEOSOME**
